# Structure and substrate specificity determinants of NfnB, a dinitroaniline herbicide–catabolizing nitroreductase from *Sphingopyxis* sp. strain HMH

**DOI:** 10.1016/j.jbc.2021.101143

**Published:** 2021-08-30

**Authors:** Sang-Hoon Kim, Sangyun Park, Eunyoung Park, Jeong-Han Kim, Sunil Ghatge, Hor-Gil Hur, Sangkee Rhee

**Affiliations:** 1 Department of Agricultural Biotechnology, Seoul National University, Seoul, Republic of Korea; 2 School of Earth Sciences and Environmental Engineering, Gwangju Institute of Science and Technology (GIST), Gwangju, Republic of Korea; 3 Research Institute of Agriculture and Life Sciences, Seoul National University, Seoul, Republic of Korea

**Keywords:** crystal structure, type I bacterial nitroreductases, NfnB, PnbA subgroup, butralin, enzyme kinetics, substrate specificity, PDB, Protein Data Bank, PNR, pendimethalin nitroreductase, TNT, 2,4,6-trinitrotoluene

## Abstract

Nitroreductases are emerging as attractive bioremediation enzymes, with substrate promiscuity toward both natural and synthetic compounds. Recently, the nitroreductase NfnB from *Sphingopyxis* sp. strain HMH exhibited metabolic activity for dinitroaniline herbicides including butralin and pendimethalin, triggering the initial steps of their degradation and detoxification. However, the determinants of the specificity of NfnB for these herbicides are unknown. In this study, we performed structural and biochemical analyses of NfnB to decipher its substrate specificity. The homodimer NfnB is a member of the PnbA subgroup of the nitroreductase family. Each monomer displays a central α + β fold for the core domain, with a protruding middle region and an extended C-terminal region. The protruding middle region of Val75–Tyr129 represents a structural extension that is a common feature to members of the PnbA subgroup and functions as an opening wall connecting the coenzyme FMN-binding site to the surface, therefore serving as a substrate binding site. We performed mutational, kinetic, and structural analyses of mutant enzymes and found that Tyr88 in the middle region plays a pivotal role in substrate specificity by determining the dimensions of the wall opening. The mutation of Tyr88 to phenylalanine or alanine caused significant changes in substrate selectivity toward bulkier dinitroaniline herbicides such as oryzalin and isopropalin without compromising its activity. These results provide a framework to modify the substrate specificity of nitroreductase in the PnbA subgroup, which has been a challenging issue for its biotechnological and bioremediation applications.

Nitroreductase is an FMN-dependent enzyme that mediates its catalysis *via* a ping–pong bi–bi reaction mechanism (1). The enzyme typically catalyzes the reduction of nitro groups in nitroaromatic and nitroheterocyclic compounds to amino or hydroxylamino groups (1). In the first of two sequential reactions, the reduced form of NADH or NADPH serves as an electron donor and reduces the coenzyme FMN with concurrent release of NAD(P)^+^, whereas in the second reaction, reduced FMN is responsible for the reduction of nitro groups in the substrate through electron transfer. Two types of nitroreductases from *Escherichia coli* were historically recognized in nitroreduction of nitrofurazone: oxygen-insensitive (type I) and oxygen-sensitive (type II) enzymes, which exhibit two-electron and single-electron transfer, respectively (2, 3). To date, members of the nitroreductase family have been found to comprise more than 20,000 sequences from bacterial, archaeal, and eukaryotic organisms (4). Beause of the rapid expansion of this family, a recent classification divided its members into 22 subgroups based on sequence, structure, phylogeny, and functional similarity network (5). Among these subgroups, eight remain to be functionally characterized. Two representatives, NfsA (6) and NfsB (7) from *E. coli*, have been adopted as templates for phylogenetic and sequence-based classification of oxygen-insensitive bacterial nitroreductases and now belong to their own NfsA and NfsB subgroups within the newly developed classification, containing 2299 and 2632 members, respectively (5).

The physiological roles of nitroreductases remain unknown, but these versatile enzymes with broad substrate specificity exhibit catalytic activity toward both natural and synthetic compounds. Therefore, nitroreductases are highly attractive for their biotechnological applications in bioremediation (8), biocatalysis (1, 9), and prodrug activation gene therapy for cancer treatment (10). In bioremediation, bacteria containing nitroreductases with the desired activity are employed to degrade or biotransform toxic and persistent nitroaromatic compounds that are produced by anthropogenic activities or industrial processes (11). For example, 2,4,6-trinitrotoluene (TNT), one of the most widely used explosives, causes soil and groundwater contamination. Consequently, TNT has become a target for bioremediation using nitroreductases (12, 13). Transgenic plants harboring bacterial genes, including pentaerythritol tetranitrate reductase, nitroreductase, or cytochrome P450, have become tolerant to TNT, and phytoremediation has been proposed for detoxifying nitroaromatic explosives. More examples of remediation using bacterial nitroreductase have been reviewed extensively elsewhere (8, 14, 15).

Recently, two bacterial nitroreductases have been shown to metabolize dinitroaniline herbicides (Fig. 1*A*): pendimethalin nitroreductase (PNR) from *Bacillus subtilis* Y3 (16, 17) and NfnB from *Sphingopyxis* sp. strain HMH (18). Dinitroaniline herbicides, of which annual consumption accounts for about 1.5% of global herbicide application (19), are nitroaromatic compounds that are hazardous to environmental safety and human health (11, 20). PNR and NfnB are oxygen-insensitive type I bacterial nitroreductases, with a low sequence identity of ∼18%, and are classified into the NfsB and PnbA subgroups, respectively. PNR, which employs both NADH and NADPH, is applicable for the nitroreduction of a broad range of dinitroaniline herbicides, with similar enzyme efficiency (*k*_cat_/*K*_*m*_) (16, 17). Specifically, PNR nitroreduction was identified only at the C6-nitro group of pendimethalin, but both at nitro groups at C2 and C6 of butralin, oryzalin, and isopropalin (Fig. 1*A*). By contrast, NfnB exhibited narrow substrate specificity toward butralin and performs two sequential reactions including nitroreduction at one nitro group and *N*-dealkylation, possibly *via* hydroxylation on the carbon of the C1 attachment group, resulting in three different metabolites (18). These reactions are the initial steps of degradation and detoxification of dinitroaniline herbicides, which are persistent in the environment, thus PNR and NfnB are promising enzymes for bioremediation.

**Figure 1:**
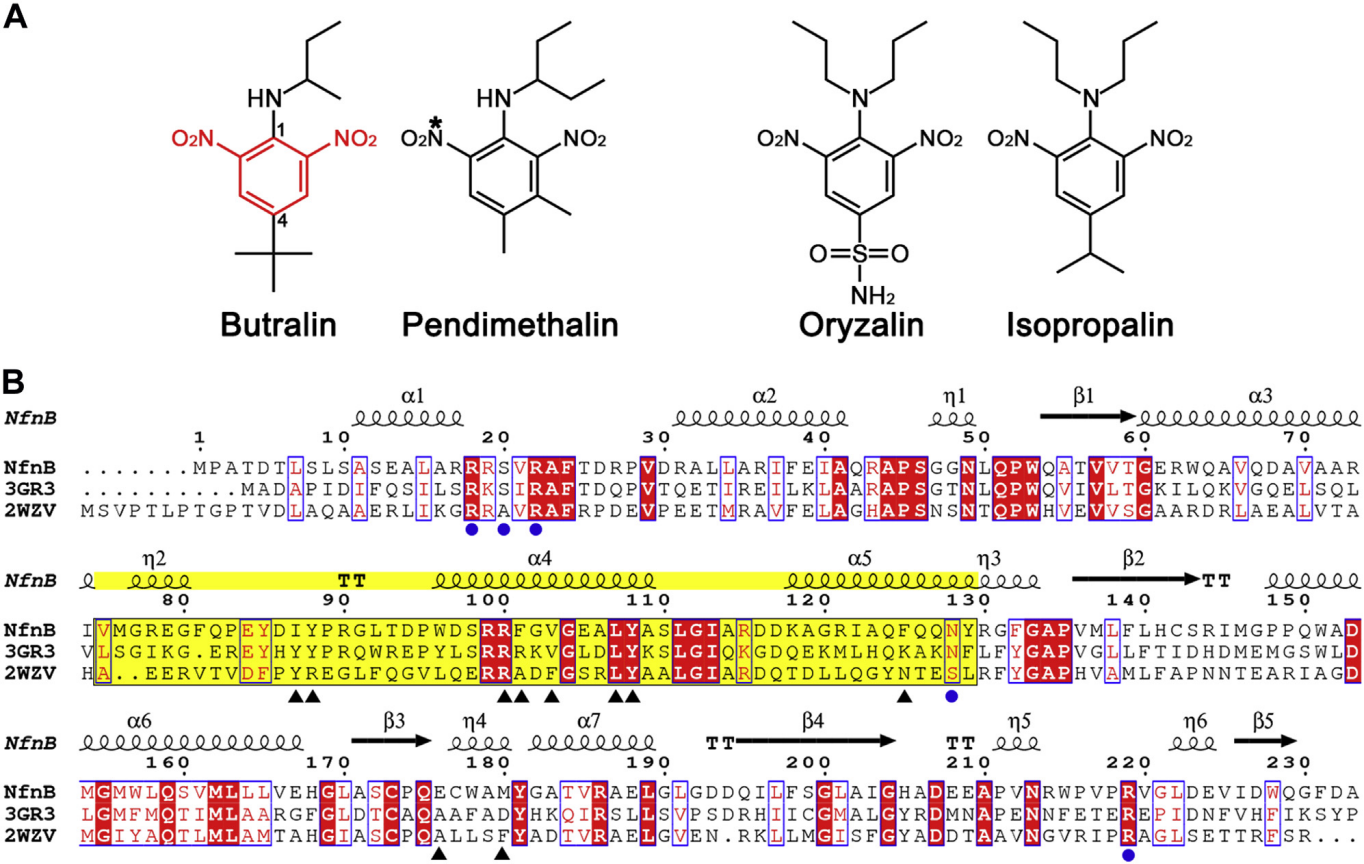
**Chemical structures of dinitroaniline herbicides and sequence alignment of NfnB.***A*, chemical structures of four dinitroaniline herbicides. The 2,6-dinitroaniline skeleton is indicated in *red*, with atoms C1 and C4 indicated. The nitro group (∗) in pendimethalin was subjected to reduction by PNR. *B*, the amino acid sequence of NfnB was compared by pairwise alignment with two structural homologs in the PnbA subgroup: nitroreductases from *Bartonella henselae* strain Houston-1 (Protein Data Bank ID: 3GR3) and *Mycolicibacterium smegmatis* MC2 155 (Protein Data Bank ID: 2WZV) (23). Highly conserved residues are indicated in *red* and *boxed* in *blue*; strictly conserved residues are shown on a *red background*. Secondary structures defined in NfnB are shown for the corresponding sequences. *Blue circles* indicate FMN binding residues; *black triangles* indicate opening wall residues; *yellow background* indicates the protruding middle region of Val75–Tyr129. This figure was prepared using the ESPript software (33). PNR, pendimethalin nitroreductase.

Elucidating the determinant(s) and modulation of substrate specificity is an ongoing challenge in the biotechnological application of nitroreductases. In the present study, we determined the crystal structure of NfnB and performed kinetic analyses to identify possible substrate specificity determinants. Our results provide a structural basis for the substrate specificity of NfnB and could be a platform to modify the substrate specificity of nitroreductases within the PnbA subgroup.

## Results and discussion

### Overall structure of NfnB

We determined the crystal structure of NfnB in the presence of coenzyme FMN at a resolution of 2.1 Å (Table 1). Two monomers in the asymmetric unit are related by noncrystallographic twofold symmetry, with a buried surface area of 7006 Å^2^, calculated using the PISA analysis (21). Given that NfnB has a monomeric molecular weight of ∼25.8 kDa and an elution peak corresponding to ∼50 kDa according to size-exclusion chromatography (Fig. S1), the dimer in an asymmetric unit likely represents a biological functional unit, that is, the homodimer characteristic of most nitroreductases (5).

**Table 1:** Data collection and refinement statistics

Dataset	Wildtype	NfnB	NfnB
	NfnB	Y88A	Y88F
PDB ID	7DP0	7DP1	7DP2
Data collection			
Wavelength (Å)	0.97933	0.97934	0.97933
Resolution (Å)	50.0–2.10 (2.18–2.10)^a^	50.0–2.00 (2.07–2.00)	50.0–2.40 (2.49–2.40)
Unique reflections	26,605	29,391	17,096
Multiplicity	13.4 (13.4)	12.9 (13.0)	14.0 (14.0)
Completeness (%)	99.9 (100.0)	99.9 (100.0)	99.9 (100.0)
Mean I/sigma(I)	12.7 (1.5)	19.4 (1.3)	20.2 (1.3)
Wilson *B*-factor (Å^2^)	37.7	36.9	57.7
*R*-merge^b^	0.27 (1.91)	0.16 (2.35)	0.17 (2.35)
CC_1/2_^c^	1.00 (0.43)	0.99 (0.54)	0.99 (0.45)
Space group	*P4*_*3*_*2*_*1*_*2*	*P4*_*3*_*2*_*1*_*2*	*P4*_*3*_*2*_*1*_*2*
Unit cell *a*, *b*, *c* (Å)	90.6, 90.6, 101.6	90.8, 90.8, 102.2	90.4, 90.4, 101.7
α, β, γ (º)	90.0	90.0	90.0
Refinement			
*R*_*work*_^d^ (%)	17.9	20.0	18.5
*R*_*free*_^e^ (%)	23.3	25.7	25.7
No. of atoms	3631	3486	3520
Macromolecules	3492	3367	3449
Ligands	62	62	62
Water	77	57	9
RMS (bonds) (Å)	0.009	0.008	0.009
RMS (angles) (º)	1.15	1.21	1.27
Ramachandran			
Favored (%)	98.2	98.4	97.7
Outliers (%)	0.00	0.00	0.00
Average *B*-factor (Å^2^)			
Macromolecules	46.4	53.5	68.9
Ligands	29.4	36.5	47.8
Water	39.7	46.5	59.3

a Numbers in parentheses refer to data in the highest resolution shell.

b *R*_*merge*_ = Σ|I_*h*_ − <I_*h*_>|/ΣI_*h*_, where I_*h*_ is the observed intensity and <I_*h*_> is the average intensity.

c The CC_1/2_ is the Pearson correlation coefficient (CC) calculated from each subset containing a random half of the measurements of unique reflection.

d *R*_*work*_ = Σ||*F*_obs_| − |*F*_cal_||/Σ|*F*_obs_|.

e *R*_*free*_ is the same as *R*_*work*_ for a selected subset (5%) of the reflections that was not included in prior refinement calculations.

**Figure 2:**
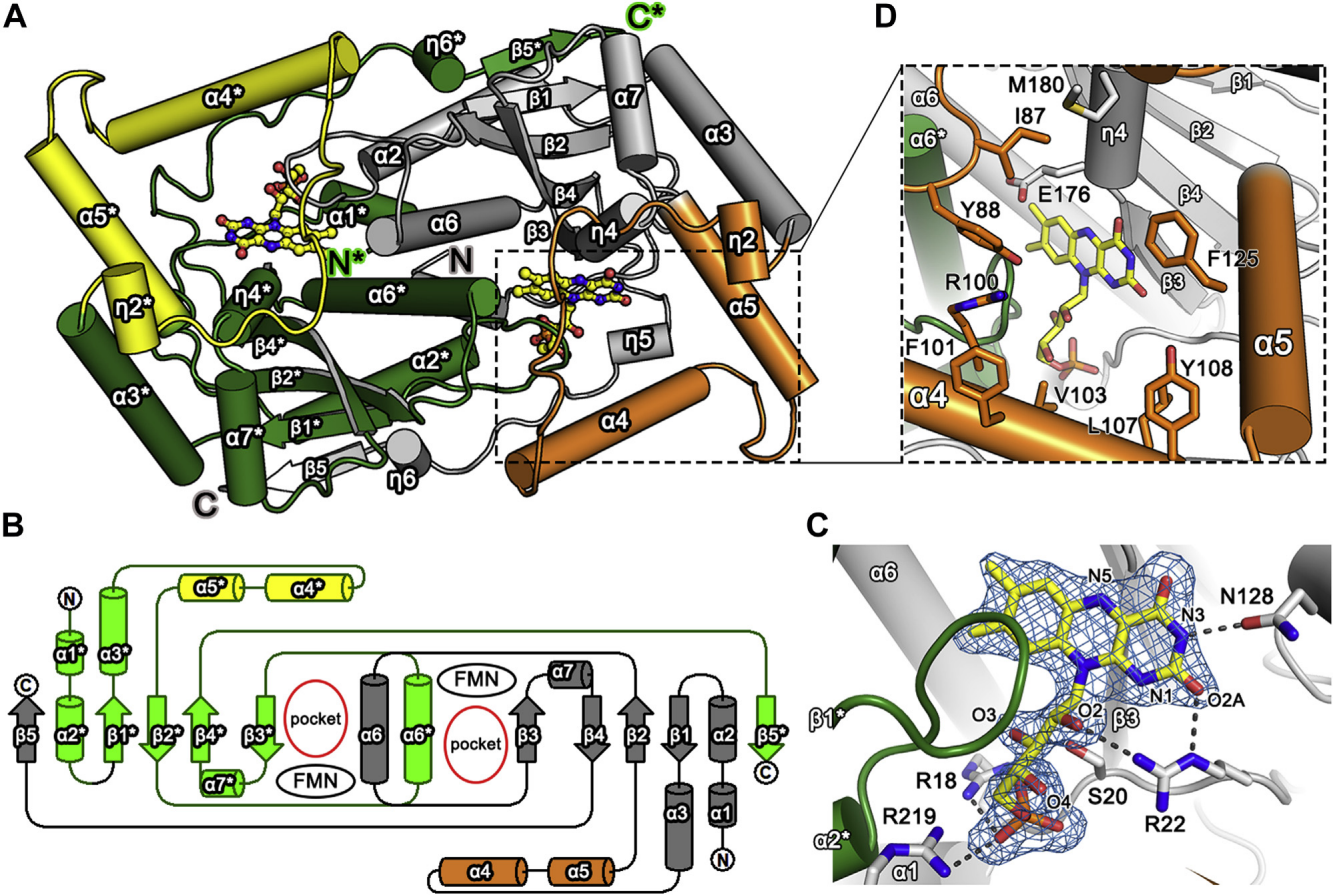
**Structure and topology of NfnB.***A*, dimeric structure of NfnB including FMN in a ball-and-stick model. The central α + β core domains and the protruding middle regions of two monomers are indicated in *gray*–*orange* and *green*–*yellow*, respectively. Labels indicate the secondary structural elements defined in Figure 1*B*. *Asterisks* indicate residues or elements from adjacent monomers. *B*, topological diagram of a homodimeric NfnB. *C*, structural environments of the FMN binding site. Coenzyme FMN is overlaid with an omitted *F*o–*F*c map contoured at 3.0 σ. *D*, enlarged view of the opening wall and its side chains and the FMN binding site. Color codes in (*B*–*D*) are as described in (*A*).

NfnB adopts an α + β fold, which is typical of the nitroreductase family of proteins (1, 5). Each monomeric NfnB, with seven α-helices and five β-strands, consists of three structural segments (Figs. 1*B* and 2*A*): a large central core domain and two extended segments from the central domain. The two extensions include a protruding middle region for Val75–Tyr129 and a 45-Å fully extended C-terminal region following Ala210. One of the helices in the protruding middle region, α4, is clustered with the C-terminal region (Fig. 2, *A* and *B*). Therefore, each monomer contains a wide concave opening at the site between the central core and the two protruding segments.

The dimerization of NfnB is achieved by facing the core domains from the two monomers in a twofold symmetric manner, with the extended C-terminal segment extensively interacting with the core domain of the neighboring monomer (Fig. 2, *A* and *B*). In the dimer, edge β1 from the central β-sheet (*i.e.*, β3–β4–β2–β1) in the core domain is further aligned with the fifth β-strand of β5∗ (the asterisk indicates a residue or an element from an adjacent monomer) from the C-terminal region of the neighboring monomer, constituting a five-stranded β-sheet. In each monomer, the central β-sheet is flanked by four α-helices: α2, α3, α6, and α7. Among these, α3 and α7 are positioned on the outer layer of the enzyme, with their helical axes almost orthogonal to those of the central β-strands and antiparallel to each other. The α4 and α5 helices in the protruding middle region are also members of the outer layer. From a topological perspective, helices α4 and α5 are not closely associated with the central β-sheet but extend by ∼15 and 23 Å, respectively, from edge β3 of the central β-sheet. The protruding middle region of Val75–Tyr129 involves a 20-residue loop following α3 as well as helices α4 and α5. These structural elements exhibit an unusual rectangular arrangement in a top view (Fig. 2*A*), generating a concave space surrounded by the central core domain and these elements. The concave space of the monomer is surrounded mainly by three structural walls, one formed by β3 from the central β-sheet, one by α5 in the protruding middle region, and one that combines with α4 and the C-terminal region (Fig. 2, *A* and *B*). Within 5 to 8 Å from the Cα atoms, the fully extended C-terminal region runs in an antiparallel direction to α4, forming a possible structural wall of the concave space. The remaining two flanking helices of the central β-sheet, α2 and α6, are parallel to the central β-strands and located at the dimer interface, resulting in four continuous helices, α2–α6–α6∗–α2∗, in the dimer, which act as dimerization elements. In particular, α6 and α6∗ mediate extensive interactions with each other, stabilizing dimeric NfnB, and also serve as the fourth structural wall of the concave space (Fig. 2, *A* and *B*). Thus, symmetrical arrangements of the two monomers effectively seal off the concave opening of the monomer, resulting in a pocket for FMN binding in the dimer interface.

### Structural comparisons with other nitroreductases

A structural similarity search program DALI (22) was run using monomeric NfnB as a search model. The results revealed two highly similar NfnB homologs that also form dimers as biological functional units and include the protruding middle region: a nitroreductase from *Bartonella henselae* strain Houston-1 (Protein Data Bank [PDB] ID: 3GR3; Z-score of 30.1; sequence identity, 34%; and RMSD of 1.36 Å for 219 Cα atoms) with yet unknown function, and another from *Mycolicibacterium smegmatis* MC2 155 (PDB ID: 2WZV; Z-score of 29.4; sequence identity, 34%; and RMSD of 1.34 Å for 214 Cα atoms) showing reductase activity for benzothiazinone, an antitubercular drug candidate (23). The dimeric structures of NfnB and the two structural homologs all belong to the PnbA subgroup, which includes more than 1400 members (Fig. 1*B*) (5). The nitroreductase family members, which are typically homodimeric enzymes, exhibits sequence variation and structural diversity. In particular, structural diversity is characterized by the insertion of an extension element into the central α + β fold at three different insertion sites (5). There are three types of extensions in the family that vary in length, sequence, and secondary structure. The protruding middle region of NfnB represents an extension in the PnbA subgroup (Fig. 1*B*), and its insertion into the loop following α3 is a feature common to the PnbA subgroup (Fig. 2, *A* and *B*). In the NfsB subgroup, a 34-residue extension is present at the loop following β2 (Fig. S2), and a ∼60-residue extension in the NfsA subgroup is located at the C terminus following a terminal β5 (Fig. S3) (24, 25). These distinct features demonstrate that structural topologies of the nitroreductases family, including the overall structure and active site environment, are diverse because of the presence of extension elements and their relative locations, which suggests that nitroreductase substrate specificity may be influenced by these extensions.

### FMN binding site in NfnB

Coenzyme FMN is bound to the pocket in the dimeric interface, with an opening to the surface. This pocket is further enclosed by a loop between α2∗ and β1∗, and η4 for 3_10_-helix, both very close to the isoalloxazine ring of FMN; a loop following α1 forms the bottom of the pocket (Fig. 2, *C* and *D*). Within the pocket, coenzyme FMN is oriented with its isoalloxazine ring packed against the β3 backbone atoms and exposed to solvent *via* the opening to the surface. The ribityl tail of FMN stretches into the inner side of the pocket, with its phosphate group near the bottom. Arg18, Ser20, Arg22, and Arg219 mediate interactions within 3.3 Å along the ribityl moiety as follows: Arg22 to O2 of the moiety, Ser20 to O3, and Arg18, Ser20, and Arg219 all to the phosphate group of the moiety (Fig. 2*C*). These tail-interacting residues are highly conserved in the PnbA subgroup of nitroreductases (Fig. 1*B*). Except for these possible hydrogen bonds along the ribityl tail, there are extensive interactions within ∼4 Å of the FMN isoalloxazine ring.

The FMN isoalloxazine ring is near η4 at the inner side of the pocket, located ∼7 Å deep from the opening (Fig. 2*D*). Except for a contribution by Met180 from η4, the opening wall consists mainly of side chains of residues in the protruding middle region, including Ile87, Tyr88, Arg100, Phe101, Val103, Leu107, Tyr108, and Phe125. Near the entrance, the opening has ∼16 Å of Cα atoms between Tyr88 and Phe125 and a similar distance between Glu176 and Val103. Therefore, the side-chain conformations of the opening wall residues and their chemical identities directly affect the dimensions and electrostatic features of the opening wall and possibly interactions with the substrate, suggesting that an extension element is involved in substrate specificity.

### Kinetic analyses and substrate specificity of the wildtype NfnB

**Figure 3:**
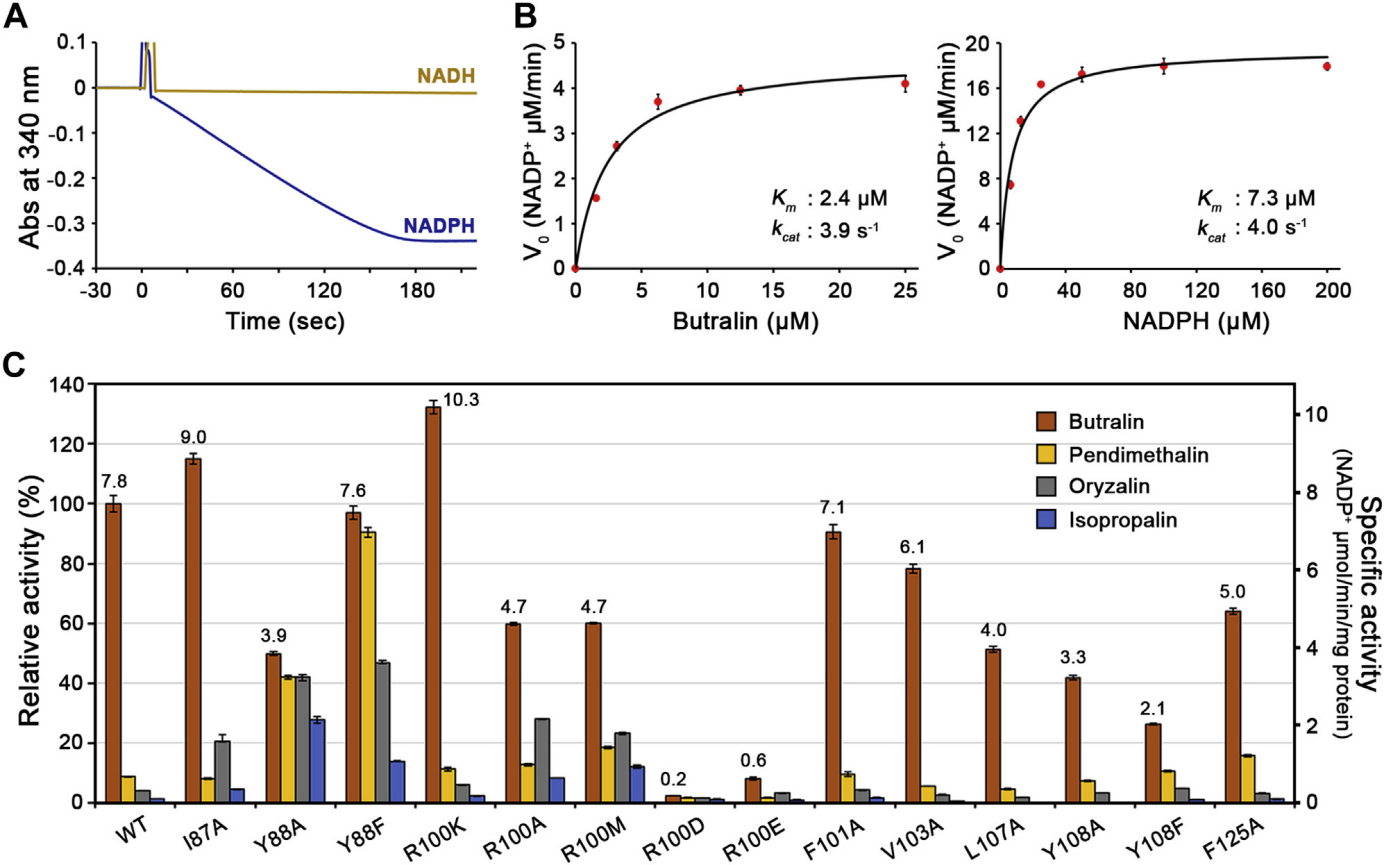
**Functional analyses of NfnB.***A*, enzyme reaction curve. Enzyme assays were performed in the presence of 50 μM NADH or NADPH and 25 μM butralin; the reaction was triggered by adding 80 nM wildtype NfnB at reaction time of 0. *B*, steady-state kinetic assays of the wildtype NfnB toward butralin and NADPH. To measure the kinetic parameters toward butralin, we used 20 nM enzyme at a saturating concentration of 50 μM NADPH; for those toward NADPH, we used 80 nM enzyme at a saturating concentration of 25 μM butralin. Each measurement was conducted in triplicate; error bars indicate SD. *C*, specific activities for NfnB and various mutants toward butralin, pendimethalin, oryzalin, and isopropalin. The reactions are described in detail in the Experimental procedures section. All measurements were compared with the specific activity of the wildtype NfnB toward butralin (*i.e.*, 7.8 NADP^+^ μmol min^−1^ mg^−1^ protein). The specific activity of each mutant for butralin is indicated. Error bars represent the SD of triplicate measurements.

We carried out steady-state kinetic analyses of the wildtype NfnB toward four dinitroaniline herbicides: butralin, pendimethalin, oryzalin, and isopropalin. First, we confirmed that NfnB employs NADPH, not NADH, as an electron donor in the first step of the reaction in a ping–pong bi–bi mechanism conserved for the nitroreductase family (Fig. 3*A*). The *K*_*m*_ and *k*_cat_ values of butralin are 2.4 μM and 3.9 s^−1^, respectively, whereas those of NADPH are 7.3 μM and 4.0 s^−1^ (Fig. 3*B*). Consistent with the findings of a previous study (18), butralin was the best substrate for the wildtype NfnB, followed by pendimethalin, showing 21-fold lower enzyme efficiency (*k*_cat_/*K*_*m*_) than butralin, whereas no activity was observed against oryzalin or isopropalin (Table 2). These four herbicides could be classified into two groups based on the size of substituents at the amino group in the C1 position: one for butralin and pendimethalin, and the other for oryzalin and isopropalin, which have bulkier substituents (Fig. 1*A*). Therefore, NfnB prefers dinitroaniline herbicides that have smaller substituents at the corresponding position.

**Table 2:** Kinetic parameters of NfnBs

Substrate	Construct	*K*_*m*_ (μM)	*k*_cat_ (s^−1^)	*k*_cat_/*K*_*m*_ (s^−1^ μM^−1^)
NADPH^a^	Wildtype	7.3 (1.4)^b^	4.0 (0.16)	5.5 × 10^−1^ (100%)^c^
	Y88A	19 (3.0)	2.2 (0.078)	1.1 × 10^−1^ (20%)
	Y88F	4.2 (0.48)	4.0 (0.080)	9.2 × 10^−1^ (167%)
	R100K	1.7 (0.18)	3.6 (0.058)	2.1 (382%)
	R100A	58 (4.5)	3.9 (0.095)	6.8 × 10^−2^ (12%)
	R100M	95 (20)	4.9 (0.30)	5.2 × 10^−2^ (9%)
Butralin^d^	Wildtype	2.4 (0.50)	3.9 (0.22)	1.6 (100%)^c^
	Y88A	0.59 (0.077)	2.1 (0.055)	3.6 (225%)
	Y88F	0.81 (0.12)	4.0 (0.12)	4.9 (306%)
	R100K	3.9 (0.73)	4.1 (0.33)	1.0 (63%)
	R100A	3.6 (1.0)	4.4 (0.41)	1.2 (75%)
	R100M	4.3 (1.3)	6.1 (0.69)	1.4 (88%)
Pendimethalin^d^	Wildtype	6.8 (1.7)	0.51 (0.038)	7.6 × 10^−2^ (100%)^c^
	Y88A	2.9 (0.18)	2.5 (0.049)	8.5 × 10^−1^ (1118%)
	Y88F	1.6 (0.28)	3.6 (0.19)	2.3 (3026%)
	R100K	4.4 (0.94)	0.41 (0.029)	9.3 × 10^−2^ (122%)
	R100A	5.5 (1.4)	0.73 (0.052)	1.3 × 10^−1^ (171%)
	R100M	5.0 (0.96)	0.97 (0.051)	1.9 × 10^−1^ (250%)
Oryzalin^d^	Wildtype	ND^e^	ND	ND
	Y88A	5.4 (0.66)	2.1 (0.074)	4.0 × 10^−1^ (100%)^f^
	Y88F	25 (1.9)	3.7 (0.075)	1.5 × 10^−1^ (38%)
	R100K	ND	ND	ND
	R100A	120 (14)	6.5 (0.29)	5.5 × 10^−2^ (14%)
	R100M	130 (13)	6.5 (0.25)	5.0 × 10^−2^ (13%)
Isopropalin^d^	Wildtype	ND	ND	ND
	Y88A	0.83 (0.16)	1.2 (0.069)	1.5 (100%)^f^
	Y88F	2.7 (0.17)	0.66 (0.012)	2.4 × 10^−1^ (16%)
	R100K	6.8 (0.80)	0.12 (0.0029)	1.8 × 10^−2^ (1%)
	R100A	2.2 (0.13)	0.44 (0.0051)	2.0 × 10^−1^ (13%)
	R100M	2.9 (0.15)	0.13 (0.0064)	4.4 × 10^−2^ (3%)

a Butralin concentrations used for these assays are as follows: 25, 12.5, 25, 9.4, 12.5 and 12.5 μM for the wildtype NfnB, Y88A, Y88F, R100K, R100A and R100M, respectively.

b The standard error in parentheses is calculated using SigmaPlot software.

c Enzyme efficiencies of mutants toward NADPH, butralin, and pendimethalin are calculated relative to that of the wildtype NfnB against a respective substrate.

d NADPH concentrations used for the assay are as follows: 50, 200, 100, 100, 400, and 400 μM for the wildtype NfnB, Y88A, Y88F, R100K, R100A, and R100M, respectively.

e Not detected.

f Enzyme efficiencies of mutants toward oryzalin and isopropalin are calculated relative to that of Y88A mutant.

These characteristics of NfnB are different from those of PNR (17), the enzyme efficiency of which is similar among butralin, pendimethalin, oryzalin, and trifluralin. PNR also has higher efficiency values for these substrates (10.9–11.9 μM^−1^ s^−1^) than NfnB for butralin (1.6 μM^−1^ s^−1^). However, because of differences in the assay conditions and methods, further comparisons of the kinetic parameters between NfnB and PNR were not attempted.

### Mutational analysis of the opening wall residues

The structure determination of NfnB in complex with a dinitroaniline substrate or/and NADP(H) was unsuccessful. We therefore performed mutational and kinetic analyses to evaluate the functional roles of the opening wall residues.

Various NfnB mutants for opening wall residues were constructed to identify possible residues or elements crucial for NfnB substrate selectivity. Residues within ∼11 Å from FMN were subject to mutation, including Ile87, Tyr88, Arg100, Phe101, Val103, Leu107, Tyr108, and Phe125 (Fig. 2*D*). Specific activities were measured for the wildtype NfnB and various mutants under 50 μM NADPH and 25 μM substrate, which are the saturating concentrations of each substrate for the wildtype NfnB. Compared with the wildtype NfnB, specific activity toward butralin varied by 2 to 132% among the NfnB mutants (Fig. 3*C*), reflecting the functional role of each residue toward butralin. In the wildtype enzyme, we measured marginal activities for pendimethalin, oryzalin, and isopropalin, at 8.8%, 4.1%, and 1.4% of that for butralin, respectively. This narrow substrate selectivity for butralin was maintained across all NfnB mutants, except Tyr88 and Arg100 (Fig. 3*C*). Unexpectedly, mutants of Tyr88 and Arg100 displayed unusual substrate preference and/or activity. Single mutants Y88A and Y88F showed broad substrate specificity, with relatively high activity. The specific activity of the Y88A mutant toward butralin was ∼50% of the wildtype enzyme; its specific activities toward pendimethalin, oryzalin, and isopropalin were ∼42%, ∼42%, and ∼28%, respectively, of the wildtype NfnB for butralin. The specific activity of the Y88F mutant against butralin was 97% of the wildtype enzyme, and it showed significantly enhanced activities toward pendimethalin (90%), oryzalin (47%), and isopropalin (14%) compared with the wildtype NfnB for butralin. Several mutants of Arg100, except R100D and R100E, showed activity for butralin, at about 60 to 132% of the wildtype NfnB, and slightly broad substrate selectivity but not as great as shown by Y88A and Y88F. Notably, the positive charge on the Arg100 position is not essential for activity but could not be replaced with a negative charge, which essentially inactivates enzymes, as characterized in R100D and R100E (Fig. 3*C*).

**Figure 4:**
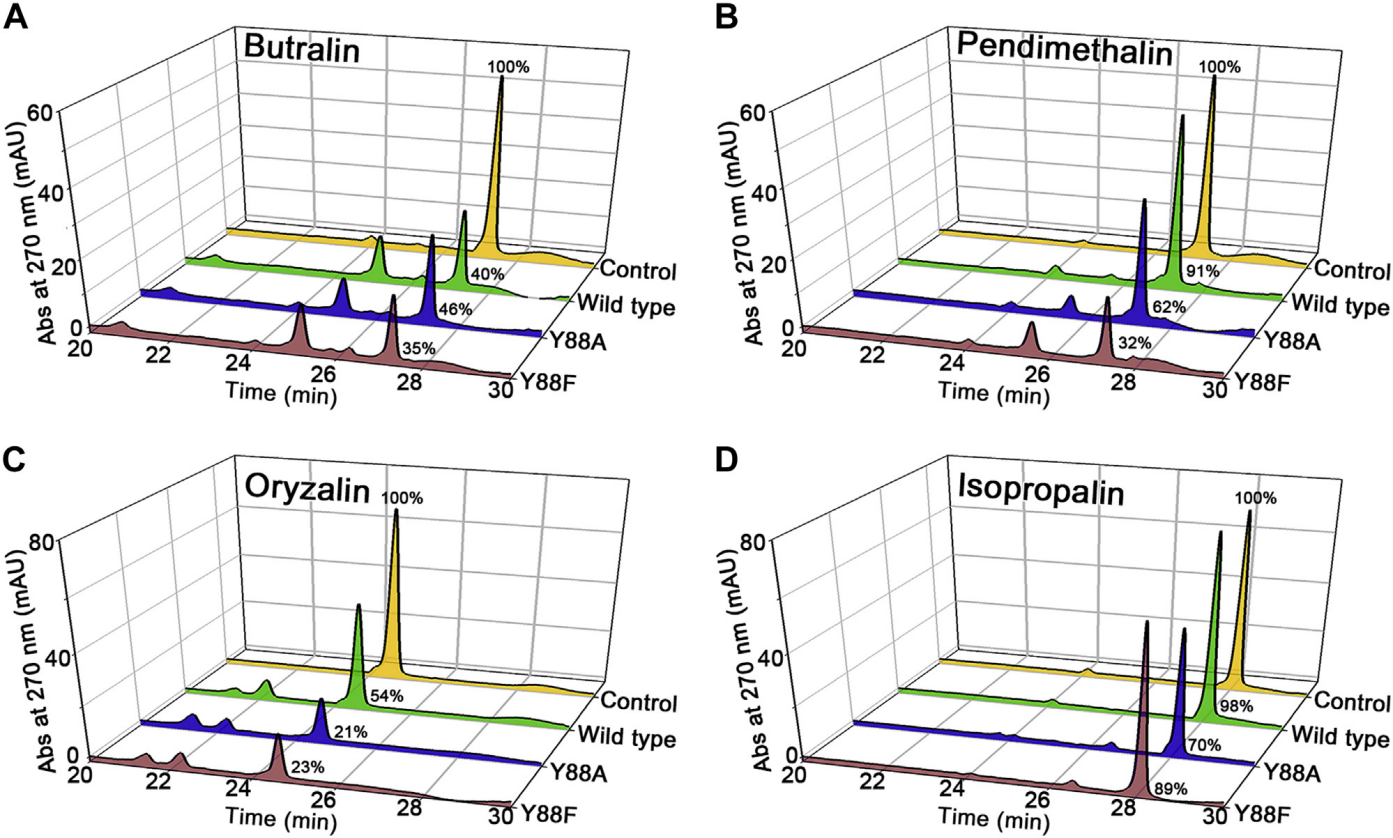
**HPLC analysis of the enzyme reaction.** HPLC chromatograms of (*A*) butralin, (*B*) pendimethalin, (*C*) oryzalin, and (*D*) isopropalin show residual amounts of reactants and products after a 5-min reaction with the respective enzyme including wildtype NfnB (*green*), Y88A (*blue*), and Y88F (*red*). The areas of the HPLC chromatograms were compared with the control (*yellow*), which represents a 0-min reaction with wildtype NfnB. In HPLC analysis with triplicate measurements, (*A*) butralin, (*B*) pendimethalin, (*C*) oryzalin, and (*D*) isopropalin were eluted at retention times of 27.3, 27.3, 24.7, and 28.2 min, respectively. The mean amount of residual substrate is indicated relative to the control.

HPLC analysis further supported our findings on the possible role of Tyr88 in substrate specificity (Fig. 4, *A*–*D*). Consistent with NfnB assays that monitor NADPH oxidation, direct measurements of the residual substrate *via* HPLC indicated that both the Y88A and Y88F mutants exhibit broad substrate specificity, with enhanced activities toward pendimethalin, oryzalin, and isopropalin. Subsequent LC–MS analysis also indicated identical catalytic reactions of butralin and pendimethalin in the Y88A mutant and the wildtype NfnB (Figs. S4 and S5) (18). We further characterized four metabolites in the Y88A mutant-dependent reaction of oryzalin and isopropalin. Even though there were no MS/MS spectra, possibly becasue of the low concentrations and lability of the metabolites, we proposed the identifications of the metabolites based on the LC–MS analysis and previously proposed oryzalin degradation pathway (Figs. S6 and S7) (26). However, further experiments, such as NMR, are required to validate the metabolites. Therefore, Tyr88 is the residue crucial for broadening substrate selectivity among dinitroaniline herbicides without significantly compromising its activity.

### Kinetic and structural analyses of Tyr88 and Arg100 mutants

**Figure 5:**
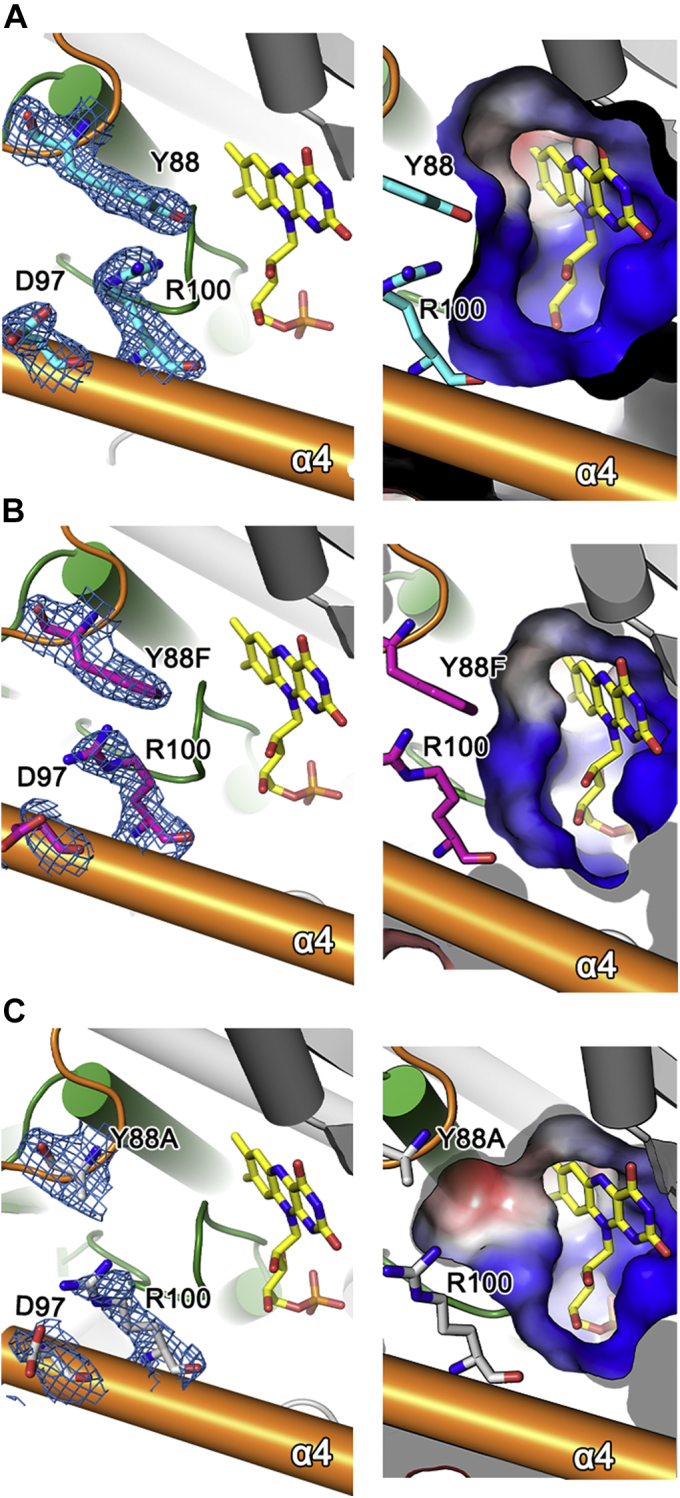
**Structural environments of Tyr88 and Arg100 in wildtype and the Y88F and Y88A mutants.** In (*A*) wildtype NfnB, (*B*) Y88F, and (*C*) Y88A, the structural environments around the opening wall are presented in an orientation similar to Figure 2*D*. *Left panels* show the FMN and side-chain conformations of Tyr88 (or Phe88 and Ala88), Asp97, and Arg100, with a 2*F*o–*F*c electron map contoured at 1.0 σ. *Right panels* show the electrostatic surface potential of the opening wall and its size. *Gray*, *red*, and *blue* indicate neutral, acidic, and basic surface conditions, respectively.

In the structure of NfnB, Tyr88 and Arg100 are neighboring residues at an entrance to the opening and show possible interaction between their side chains within ∼3.5 Å (Figs. 2*D* and 5*A*). This observation is applicable to only one specific monomer; in the other monomer, the swung-out conformation of Arg100 forms crystallographic packing interactions with a crystallographic symmetry-related Asp26 (Fig. S8). Notable changes in the substrate selectivity of Tyr88 mutants toward the four substrates suggest that the chemical identity, side-chain conformations, and/or possible interactions of these two residues could be structural determinants of NfnB substrate selectivity.

Kinetic analysis was performed on the Tyr88 and Arg100 mutants (Table 2). After comparing the efficiency of Y88A and Y88F, we concluded that both enzymes were more efficient than the wildtype NfnB, by about 2.3- and 3.1-fold, toward butralin, and by 11- and 30-fold against pendimethalin, respectively. These two mutants also exhibited high efficiency toward dinitroaniline herbicides with bulky substituents at the C1 position (*i.e.*, oryzalin and isopropalin), at 9.4 to 94% of that of the wildtype NfnB toward butralin (*i.e.*, 1.6 s^−1^ μM^−1^). In particular, Y88F exhibited higher efficiency toward butralin and pendimethalin, with a lower *K*_*m*_ value than the wildtype NfnB, but Y88A was more competent than Y88F toward oryzalin and isopropalin, mainly because of its lower *K*_*m*_ value. Unlike the Tyr88 mutation, those on Arg100, including R100K, R100A, and R100M, did not show significant changes in *K*_*m*_ and *k*_cat_ values toward butralin and pendimethalin, ranging from 65 to 179% and 80 to 190%, respectively, compared with the wildtype NfnB (Table 2). Accordingly, their enzyme efficiencies were similar to the wildtype enzyme, with a 1.1- to 1.6-fold decrease and 1.2- to 2.5-fold increase toward butralin and pendimethalin, respectively (Table 2). However, the enzyme efficiencies of Arg100 mutants to oryzalin and isopropalin were only 0 to 14% of Y88A, the more efficient of the two Tyr88 mutants toward oryzalin and isopropalin. Effects of the Arg100 mutants were also observed on the *K*_*m*_ value of NADPH (Table 2). Except for the conservative mutant R100K, the *K*_*m*_ value for NADPH increased by about 8- or 13-fold in R100A and R100M, respectively, compared with the wildtype. Therefore, Arg100 is crucial for binding NADPH, a common substrate for nitroreductase, and Tyr88 is involved more directly in substrate selectivity. Consistent with this finding, the residue corresponding to Arg100 is conserved in members of the PnbA nitroreductases (Fig. 1*B*). However, other residues in the opening wall, including Tyr88, are relatively diverse in their chemical identities among the PnbA subgroup, implying that these opening wall residues are unique to each enzyme and could be substrate specificity determinants.

Next, we determined the crystal structures of the two single mutants, Y88A and Y88F. Their structures were found to be nearly identical to the wildtype NfnB, with RMSD values of 0.38 Å for 428 Cα atoms in Y88A and 0.39 Å for 433 Cα atoms in Y88F. In the following description, we focus on the structural environments of one specific monomer, in which Tyr88 and Arg100 of the wildtype enzyme form a possible interaction. In the Y88F structure, the side chains of Phe88 and Arg100 are well ordered, at positions nearly identical to those of the wildtype NfnB (Fig. 5*B*). Compared with the wildtype NfnB, the side chain of Arg100 had been displaced by ∼4 Å toward the side chain of Asp97 near the entrance and was found within a ∼3-Å hydrogen-bonding distance from Asp97. Except for these displacements, no other residues at the entrance exhibited any noticeable conformational changes. Therefore, the possible hydrogen bond between Tyr88 and Arg100 identified in the wildtype NfnB is not crucial for maintaining their side-chain conformations or for NfnB activity (Table 2). Almost identical structural features of Arg100 were characterized in the Y88A mutant (Fig. 5*C*). Therefore, the opening wall residues Tyr88 and Arg100, which are intimately involved in enzyme activity and substrate specificity, show no dynamic features in their side chain conformations, suggesting that the static dimension of the opening and the resulting electrostatic properties are key features in the substrate specificity of NfnB.

### Substrate specificity determinants

Our structural and kinetic analyses suggested that the effects of Y88A or Y88F on substrate specificity may be due to the electrostatic properties and/or dimensions of the opening to the coenzyme FMN, that is, a possible steric hindrance against a larger substrate. Based on this conclusion and our kinetic data, we further hypothesized a possible binding mode of dinitroaniline herbicides in NfnB, such that the side chain of Tyr88 is in the immediate vicinity of hydrophobic substituents at the amino group of dinitroaniline herbicides (Fig. 1*A*): *N*-*sec*-butyl group and *N*-pentan-3-yl group for butralin and pendimethalin, respectively, and the larger *N,N*-dipropyl group for oryzalin and isopropalin. The hydroxyl group at Tyr88 appears to cause unfavorable interactions with these hydrophobic substituents. Therefore, even the absence of the side-chain hydroxyl group in the Y88F mutant unexpectedly caused significant changes of many enzymatic properties, without compromising its activity. The opening dimension appears to be a major determinant of the substrate selectivity of NfnB. Compared with the wildtype NfnB, the smaller side chain in Y88A and Y88F mutants exhibited a wider opening to the coenzyme FMN (Fig. 5, *A*–*C*). Consistent with the possible steric hindrance posed by Tyr88 in the wildtype NfnB, Y88F catalyzes enzyme reactions more efficiently for butralin and pendimethalin, and Y88A becomes competent for oryzalin and isopropalin, with larger hydrophobic substituents at the amino group. Molecular docking of oryzalin and isopropalin was analyzed using AutoDock (27) and supported the proposed functional role of Tyr88 (Fig. S9).

## Conclusion

In this study, we performed structural and kinetic analyses of NfnB from *Sphingopyxis* sp. strain HMH to decipher its substrate selectivity toward four dinitroaniline herbicides: butralin, pendimethalin, oryzalin, and isopropalin. The three-dimensional structure of NfnB is similar to other nitroreductases in the PnbA subgroup, with a protruding middle region in the loop following α3. Residues in the middle region act as opening wall to the coenzyme FMN. Kinetic analyses of various mutants in the opening wall indicated that Tyr88 is crucial for substrate selectivity. Mutants such as Y88A and Y88F broadened its substrate specificity without significantly compromising its activity. Further structural analyses suggested that Tyr88 could determine the size of the opening wall. Therefore, the mutation of Tyr88 into smaller side chains allows bulky substrates such as oryzalin and isopropalin to be efficiently catalyzed. These structural and kinetic analyses of NfnB could provide a framework for modifying the substrate specificity of nitroreductase, which has remained a challenge to its biotechnological application.

## Experimental procedures

### Cloning and purification of NfnB

The gene for NfnB from *Sphingopyxis* sp. strain HMH (National Center for Biotechnology Information Reference Sequence: WP_076073454.1) was amplified by polymerase chain reaction using a full-length gene as a template and cloned into a modified pET28b vector (Merck) containing a tobacco etch virus protease recognition site between the His_6_ tag and multicloning site. The resulting vector harboring the gene for N-terminal His-tagged NfnB was transformed into *E. coli* BL21 (DE3) (Novagen) and plated onto Luria–Bertani agar with 50 μg/ml kanamycin. Luria–Bertani culture containing *E. coli* BL21 transformed with the plasmid for NfnB was grown at 37 °C until an absorbance at 600 nm of 0.6 was attained, then the recombinant NfnB was induced by adding 0.5 mM isopropyl-β-d-thiogalactopyranoside for 14 to 16 h at 20 °C. *E. coli* cells were collected, suspended, and sonicated in buffer A (50 mM Tris [pH 8.0] and 100 mM NaCl) and 5 mM FMN. The N-terminal His-tagged NfnB was purified through immobilized metal affinity chromatography using a HisTrap HP column (GE Healthcare) with buffer A and eluted with buffer A plus 500 mM imidazole in tobacco etch virus. The resulting tag-free NfnB protein was further purified *via* immobilized metal affinity and subjected to size-exclusion chromatography using a Superdex 200 column (GE Healthcare) with buffer A.

For further functional and structural analysis, genes of various NfnB mutants were produced by site-directed mutagenesis using the QuikChange method (Agilent) with a pair of mutagenic primers (Table S1). To determine the structures of the NfnB mutants Y88A and Y88F, we followed protein purification procedures identical to those described previously.

### Crystallization and structure determination of NfnB

The tag-free wildtype NfnB and mutants Y88A and Y88F in buffer A were concentrated to 15 mg/ml. Crystallization was conducted through drop vapor diffusion at 22 °C using a crystallization solution of 100 mM Tris (pH 7.0), 40% (v/v) PEG 300, and 8% (v/v) PEG1000. Cryoprotection was achieved using 28.5% (v/v) PEG400 for the wildtype NfnB crystals and 20% glycerol for crystals of the Y88A and Y88F mutants. X-ray diffraction data were collected at 100 K, with an oscillation angle of 0.5°, on beamline 7A at the Pohang Accelerator Laboratory. Collected data were processed using the HKL2000 software (28), and a high-resolution data cutoff was determined by CC_1/2_ statistical value of ∼0.5 (29, 30). The space groups of all crystals were *P4*_*3*_*2*_*1*_*2*, which contained two monomers in the asymmetric unit.

The structure of the wildtype NfnB was determined by molecular replacement using the PHENIX software, with the structure of *Mycobacterium smegmatis* (PDB ID: 2WZW; sequence identity, 33%) (23) as a search model. Several cycles of manual modeling and refinement were performed using the COOT (31) and PHENIX programs (32), respectively. The structures of the Y88A and Y88F mutants were determined using the wildtype NfnB as a starting model. Details of the data collection and refinement processes are presented in Table 1.

### Activity assays

For activity assays, the wildtype enzyme and mutants with the N-terminal His tag were expressed and purified by immobilized metal affinity chromatography as described previously. Afterward, we recognized that enzyme activity had decreased significantly (∼50%) within 4 h, largely as a result of the buffer solution. Extensive search showed that buffer B (50 mM sodium acetate, 100 mM NaCl, 10% glycerol, and pH 5.0) was the most effective option. Therefore, the His-tagged enzymes in buffer A after immobilized metal affinity chromatography were subjected to buffer exchange with buffer B using a HiPrep 26/10 column (GE Healthcare). The resulting enzymes in buffer B were snap frozen under liquid nitrogen and stored at −80 °C. A subsequent assay showed that they retained ∼95% activity after 4 h on ice.

Enzyme assays were performed by monitoring the oxidation of NADPH to NADP^+^ in the presence of a given substrate and enzyme. Four dinitroaniline herbicides (butralin, pendimethalin, oryzalin, and isopropalin) were used as substrates, and decreases in their absorbance at 340 nm were recorded using an UV-spectrophotometer (JASCO). We confirmed that no additional FMN was necessary for the activity assay under our assay conditions. For specific activity measurement, 600 μl of reaction mixture containing 50 mM Tris (pH 7.5), 50 μM NADPH, and 25 μM substrate dissolved in dimethyl sulfoxide was incubated at 30 °C for 90 s. The reaction was then initiated by adding a given concentration of enzyme. Specifically, fixed volumes of substrate (15 μl) and enzyme (6 μl) were used to eliminate any possible effects of dimethyl sulfoxide or buffer B on enzyme activity. The initial velocity of each reaction was measured for 10 to 40 s and calculated as NADP^+^ release per minute, with an extinction coefficient of 6220 M^−1^ cm^−1^ for NADPH at 340 nm. A steady-state kinetic assay was performed in an identical manner. Assays were conducted in triplicate, and *V*_*max*_ and *K*_*m*_ were calculated using SigmaPlot (Systat Software).

### HPLC analyses

An Agilent 1100 HPLC system was used to characterize residual dinitroanilines metabolized by the wildtype NfnB and its mutants. The reaction mixture was identical to those used in the enzyme assay described previously, but with 400 μM NADPH and 200 μM substrate. After 90 s of preincubation, 500 nM of the enzyme of interest was added to the reaction mixture, followed by incubation for 5 min at 30 °C. The enzyme reaction was stopped with 1.5 ml ethyl acetate. We noticed that evaporating the solvent ethyl acetate by centrifugation in vacuum caused almost complete loss of the reactants and products. Therefore, after thorough mixing, the organic solvent containing the reactants and products was collected for analysis.

HPLC analysis was performed using an Agilent 1100 HPLC system equipped with a binary pump, autosampler, vacuum degasser, and column compartment, coupled to a diode array detector. Separation was performed using a Raptor Biphenyl column (100 × 2.1 mm, 2.7 μm, 12 nm, Raptor) at an oven temperature of 40 °C. The flow rate was 0.2 ml min^−1^, and the injection volume was 5 μl. Mobile phase A was 0.1% formic acid in water, and mobile phase B was 0.1% formic acid in acetonitrile. The gradient elution program started with 10% eluent B, increasing to 40% over 15 min, linearly increasing to 70% over 7 min, followed by a linear gradient to 100% over 3 min. The final composition was held for 10 min before returning to the initial condition over 0.1 min and then equilibrated for 14.9 min. The total run time was 50.0 min. The analytes were detected by diode array detector at 270 nm.

### LC–MS analysis

LC–MS analysis is performed to identify the metabolites produced by an enzyme-dependent reaction. The reaction conditions were identical to those of the HPLC analysis, except for a higher enzyme concentration of 1 μM and an extended reaction time of 30 min. As described for the HPLC analysis, the enzyme reaction was stopped with 1.5 ml of ethyl acetate. The organic solvent containing the reactants and products was then collected for analysis, after thorough mixing.

LC–MS analysis was performed using a Shimadzu Nexera X2 ultra-HPLC system coupled to a Shimadzu LCMS-8040 triple quadrupole mass spectrometer. The UHPLC system consisted of a degassing unit (DGU-20A5), solvent delivery module (LC-30AD), autosampler (SIL-30AC), and column oven (CTO-20A). The separation procedure was identical to those used in the HPLC analysis, with a flow rate of 0.2 ml min^−1^ and an injection volume of 5 μl.

In the MS system, ionization of the target analytes was performed in electrospray ionization positive mode. Mass spectra of the samples were obtained by scanning between *m/z* 100 and 500. The desolvation line and heat block temperatures were 250 and 400 °C, respectively. The nebulizing (nitrogen) and drying gas (nitrogen) flow rates were 3 and 15 l min^−1^, respectively. The collision-induced dissociation gas was argon. The LabSolutions LC–MS software (version 5.60; Shimadzu Europa) was used for data processing.

## Data availability

The atomic coordinates and structural factors have been deposited in the PDB (http://www.rcsb.org) under ID codes 7DP0, 7DP1, and 7DP2.

## Supporting information

This article contains supporting information (18, 26, 27, 33).

## Conflict of interest

The authors declare that they have no conflicts of interest with the contents of this article.
