# Supplementary material for: Structure and substrate specificity determinants of NfnB, a dinitroaniline herbicide–catabolizing nitroreductase from Sphingopyxis sp. strain HMH
Source: J Biol Chem. 2021 Aug 30;297(4):101143. doi: 10.1016/j.jbc.2021.101143 (PMC8484813; doi:10.1016/j.jbc.2021.101143)
Supplement: Supplemental Figures S1–S9 and Table S1 [file mmc1.pdf]

SUPPORTING INFORMATION

Structure and substrate specificity determinants of NfnB, a dinitroaniline herbicide-catabolizing nitroreductase from soil bacterium *Sphingopyxis* sp. strain HMH

Sang-Hoon Kim[1,†], Sangyun Park[1,†], Eunyoung Park[1], Jeong-Han Kim[1], Sunil Ghatge[2], Hor-Gil Hur[2], Sangkee Rhee[1,3]

[1]Department of Agricultural Biotechnology, Seoul National University, Seoul, Republic of Korea.

[2]School of Earth Sciences and Environmental Engineering, Gwangju Institute of Science and Technology (GIST), Gwangju, Republic of Korea

[3]Research Institute of Agriculture and Life Sciences, Seoul National University, Seoul, Republic of Korea.

†These two authors are equally contributed to this work

To whom correspondence should be addressed: Sangkee Rhee, Department of Agricultural Biotechnology, Seoul National University, Seoul 08826, KOREA, Fax: +82 2 8733112; Tel.: +82 2 8804647; E-mail: srheesnu@snu.ac.kr

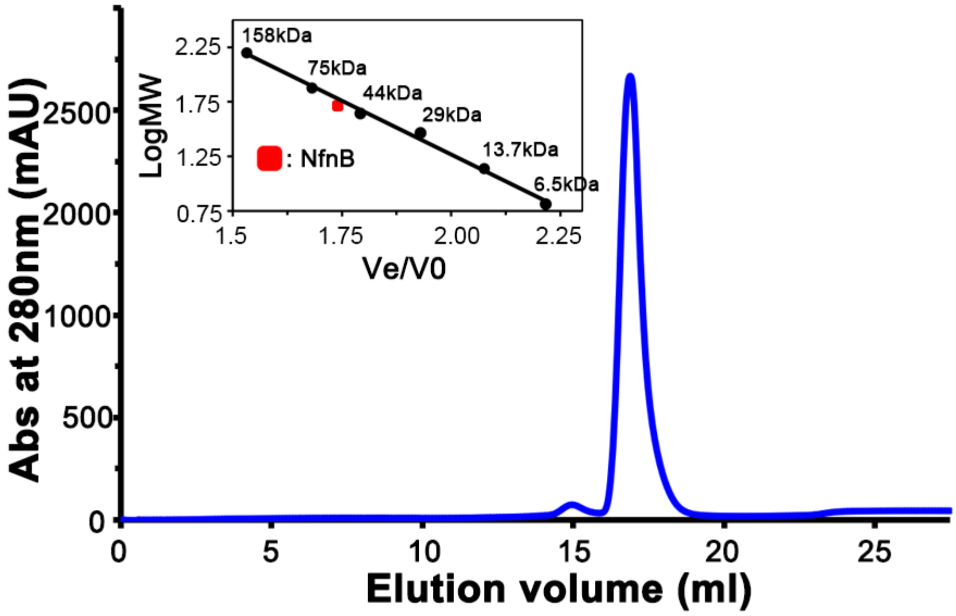

FIGURE S1. Size-exclusion chromatography analysis of NfnB. Elution profile of NfnB determined by size-exclusion chromatography. NfnB in buffer containing 50 mM phosphate (pH 7.4) and 150 mM NaCl was eluted using a Superdex 200 column (GE Healthcare). The eluted peak for NfnB was estimated to be ~50 kDa by comparison with 6.5–158 kDa molecular mass markers (GE Healthcare).

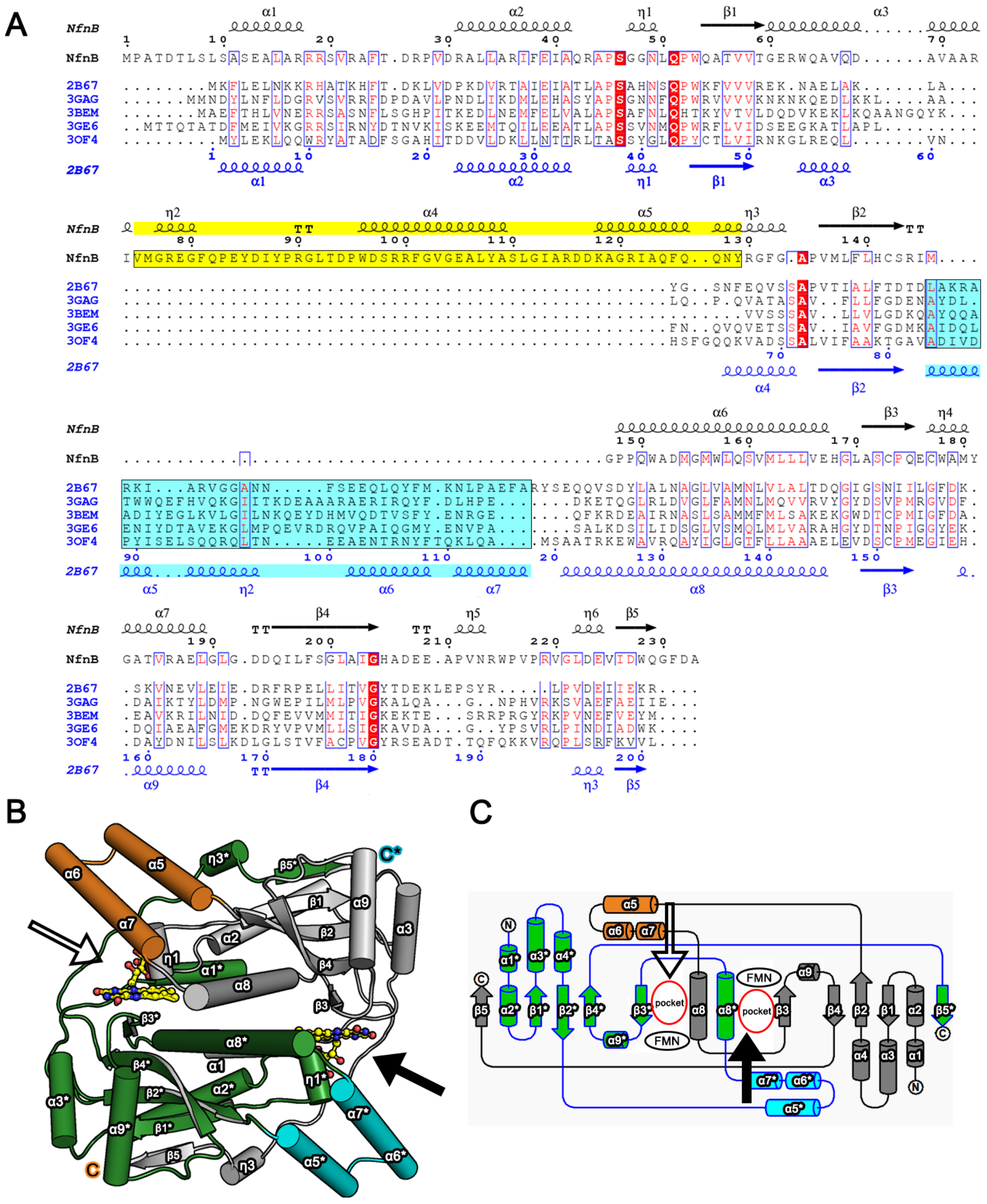

FIGURE S2. Sequence comparison of nitroreductases in the PnbA and NfsB subgroups and structural features of NfsB. (*A*) Amino acid sequences of NfnB from the PnbA subgroup were compared with those in the NfsB subgroup, which have known structures. Members of the NfsB subgroup include those from *Streptococcus pneumoniae* TIGR4 (PDB ID, 2B67; Z score, 17.9; sequence identity, 25%), *Streptococcus mutans* (PDB ID, 3GAG; Z score, 17.0; sequence identity, 22%), *Bacillus subtilis* (PDB ID, 3BEM; Z score, 16.4; sequence identity, 19%), *Exiguobacterium sibiricum 255-15* (PDB ID, 3GE6; Z score, 16.4; sequence identity, 20%) and *Idiomarina loihiensis* (PDB ID, 3OF4; Z score, 16.2; sequence identity, 14%). Highly conserved residues are indicated in red and boxed in blue. Strictly conserved residues are indicated by a red background. Secondary structural elements of NfnB

in this study and NfsB (PDB ID, 2B67) from the NfsB subgroup are shown for the corresponding sequences at the top and bottom of the sequence alignment, respectively. Extension elements of PnbA (i.e., protruding middle region of NfnB) and NfsB subgroups are indicated in yellow and cyan, respectively. Note the presence of an extension in NfsB between β2 and α8. This figure was prepared using the ESPript software (33). (*B*) Overall structure of nitroreductase (PDB ID, 2B67) in the NfsB subgroup. Color codes are as in Figure 2*A*, except for extension elements in cyan for the monomer in green, and those in orange for the monomer in gray. Arrows correspond to two active sites in a dimer at distinct locations shown in a topological view. Unlike NfnB in this study, the extension in NfsB is involved in forming the active site of the adjacent monomer. (*C*) Topological diagram of a structure from the NfsB subgroup. Color codes are as in (*B*), and the two active sites indicated by arrows correspond to those shown in (*B*).

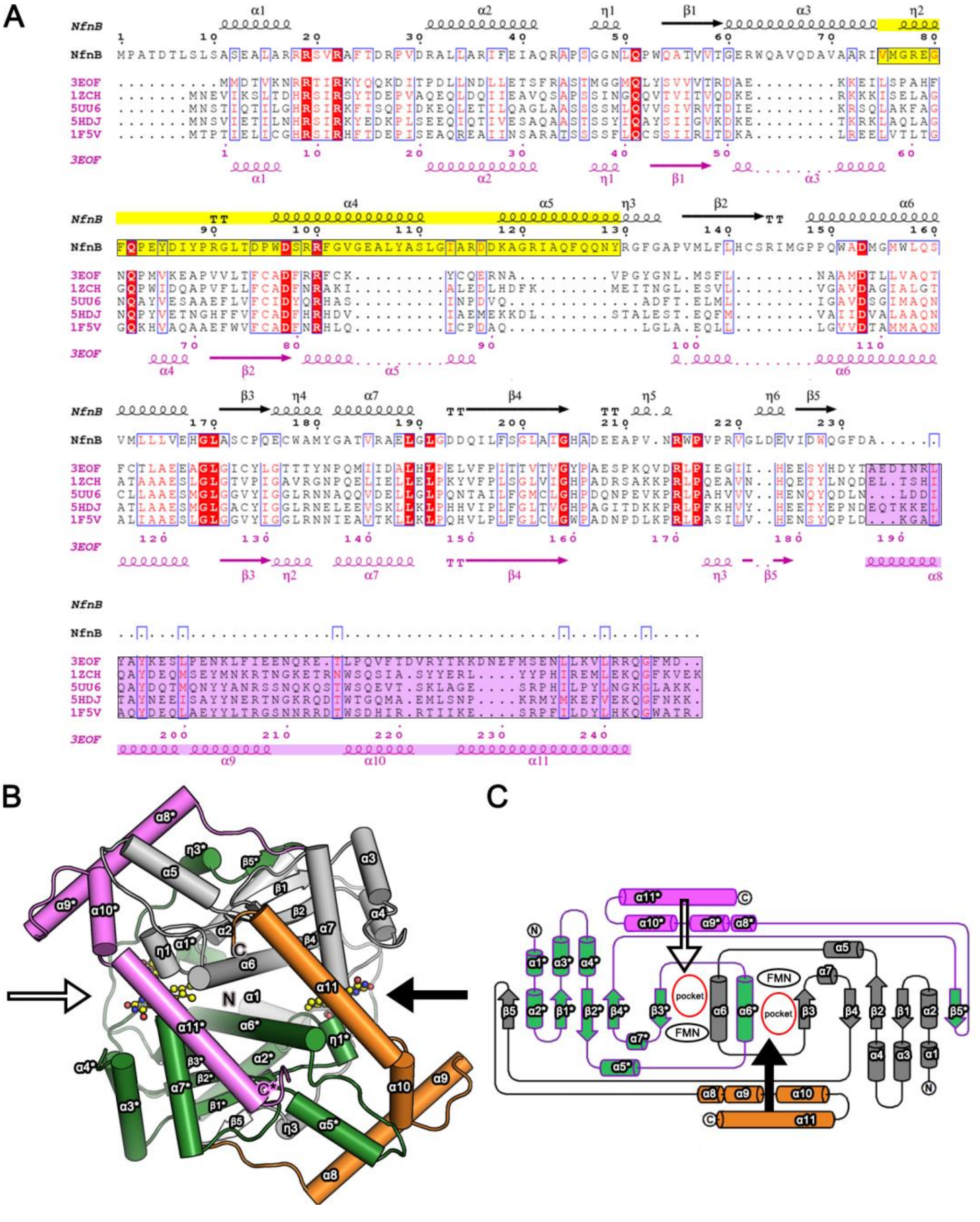

FIGURE S3. Sequence comparison of nitroreductases in the PnbA and NfsA subgroups, and structural features of NfsA. (*A*) Amino acid sequences of NfnB from the PnbA subgroup were compared with those in the NfsA subgroup, which have known structures. Members of the NfsA subgroup include those from *Bacteroides fragilis* NCTC 9343 (PDB ID, 3EOF; Z score, 16.0; sequence identity, 20%), *Bacillus subtilis* (PDB ID, 1ZCH; Z score, 15.2; sequence identity, 26%), *Vibrio parahaemolyticus RIMD 2210633* (PDB ID, 5UU6; Z score, 15.1; sequence identity, 23%), *Bacillus megaterium* (PDB ID, 5HDJ; Z score, 14.8; sequence identity, 20%) and *Escherichia coli* (PDB ID, 1F5V; Z score, 14.7; sequence identity, 19%). Secondary structural elements of the NfsA subgroup are based on the structure of NfsA (PDB ID, 3EOF). The extension elements of the NfsB subgroup (pink) are located at the C-terminal region following β5. This figure was prepared using the

ESPript software (33). (*B*) Overall structure of nitroreductase (PDB ID, 3EOF) in the NfsA subgroup. The extension element in pink belongs to the monomer indicated in green; that in orange belongs to the monomer indicated in gray. Unlike the NfsB subgroup, the C-terminal extension in NfsA is folded back and involved in forming the active site of its own monomer, not an adjacent one. (*C*) Topological diagram of a structure from the NfsA subgroup. Color codes are as in (*B*), and the two active sites indicated by arrows correspond to those in (*B*).

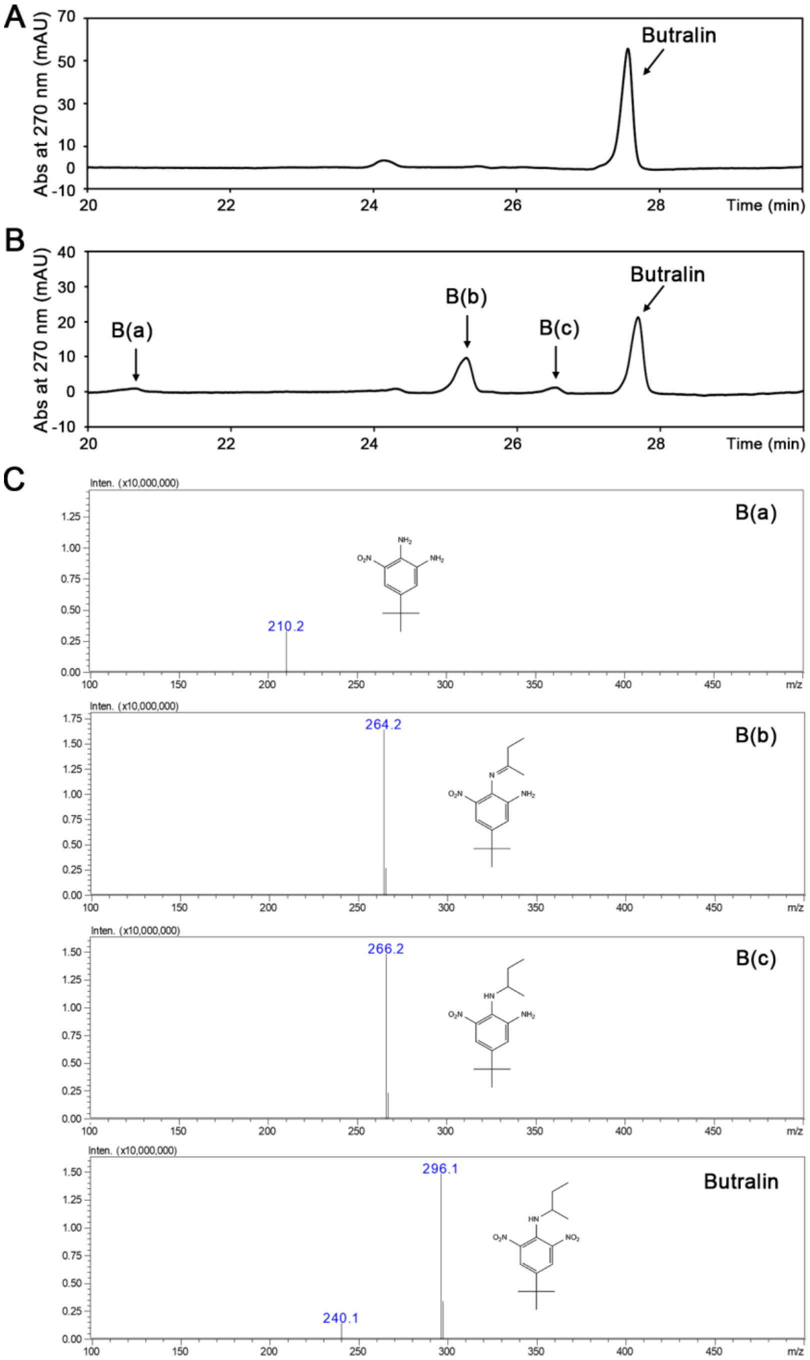

FIGURE S4. LC-MS analyses of metabolites from butralin in the Y88A mutant. (*A*) The HPLC chromatogram of butralin is shown as a standard. (*B*) The HPLC chromatogram of the Y88A-dependent reaction for 30 min shows a peak for butralin at 27.7 min, with three additional peaks, B(a), B(b), and B(c), at retention times of 20.6, 25.3, and 26.5 min, respectively. (*C*) LC-MS spectra corresponding to butralin and each metabolite. The chemical structure of each metabolite is indicated. Note that the spectra of three metabolites are consistent with those of a previous study (18).

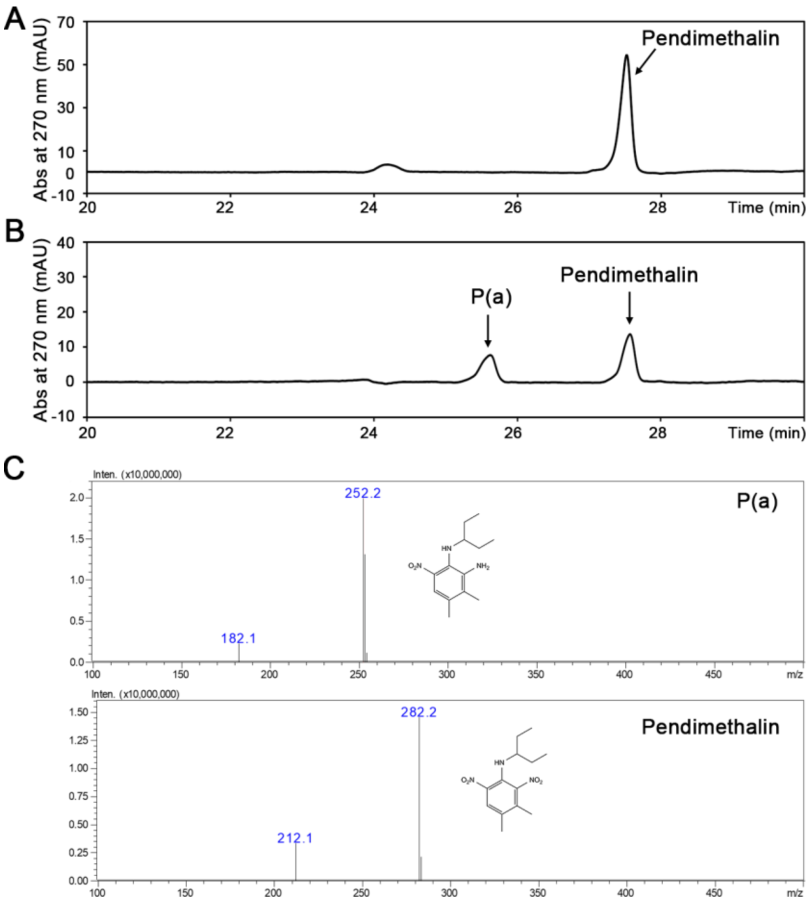

FIGURE S5. LC-MS analyses of metabolites from pendimethalin in the Y88A mutant. (*A*) The HPLC chromatogram of pendimethalin is shown as a standard. (*B*) The HPLC chromatogram of the Y88A-dependent reaction for 30 min shows a peak for pendimethalin at 27.6 min, with a new peak, P(a), produced at a retention time of 25.6 min. (*C*) LC-MS spectra corresponding to pendimethalin and its metabolite. The chemical structure of each metabolite is indicated. Note that the metabolite spectrum is consistent with that reported in a previous study (18).

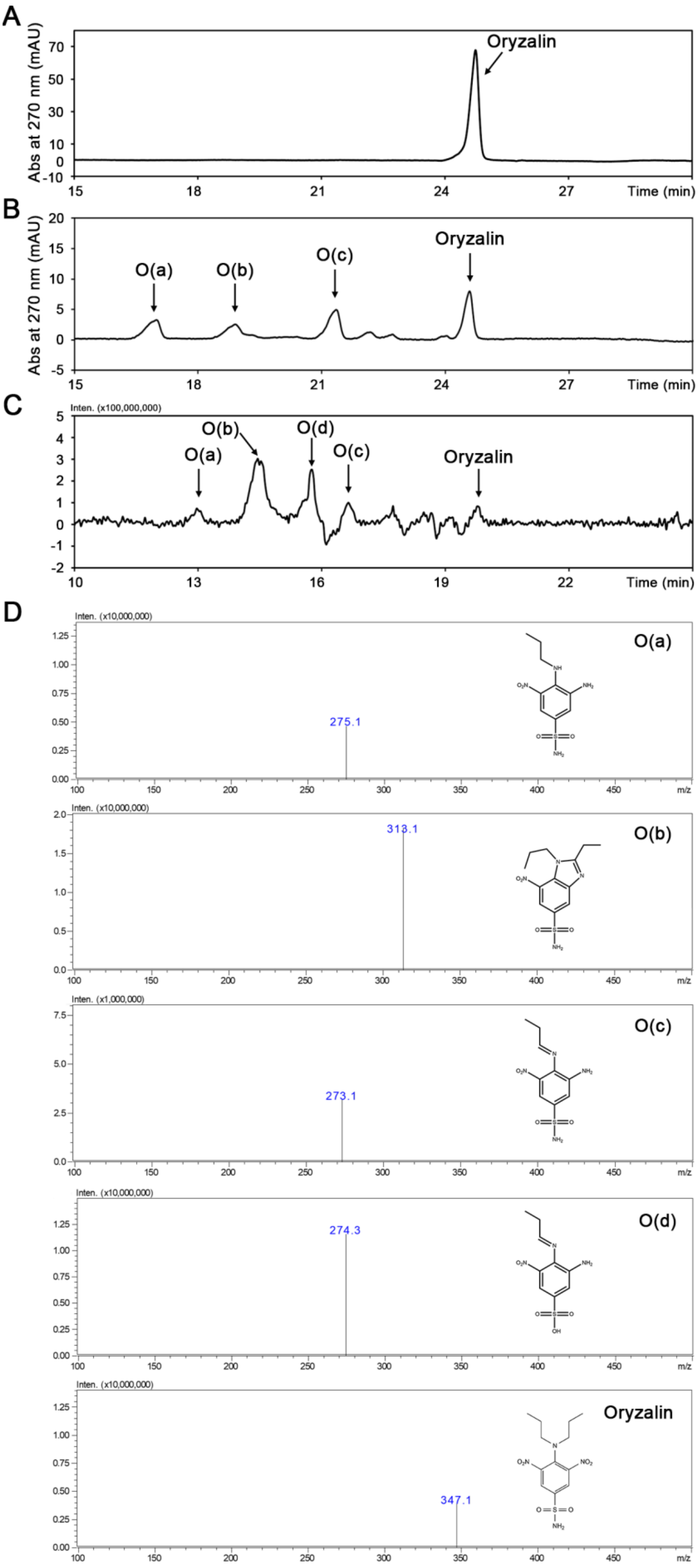

A
Oryzalin
Abs at 270 nm (mAU)
Time (min)
B
O(a)
O(b)
O(c)
Oryzalin
Abs at 270 nm (mAU)
Time (min)
C
Inten. (x100,000,000)
O(a)
O(b)
O(d)
O(c)
Oryzalin
Time (min)
D
Inten. (x10,000,000)
O(a)
275.1
m/z
Inten. (x10,000,000)
O(b)
313.1
m/z
Inten. (x1,000,000)
O(c)
273.1
m/z
Inten. (x10,000,000)
O(d)
274.3
m/z
Inten. (x10,000,000)
Oryzalin
347.1
m/z

FIGURE S6. LC-MS analyses of metabolites from oryzalin in the Y88A mutant. (*A*) The HPLC chromatogram of oryzlin is shown as a standard. (*B*) The HPLC chromatogram of the Y88A-dependent reaction for 30 min shows a peak for oryzalin at 24.6 min, with additional peaks, O(a), O(b), and O(c), at retention times of 17.0, 18.9, and 21.4 min, respectively. (*C*) Total ion chromatogram (TIC) showing another metabolite of O(d) that was not detected at an absorbance of 270 nm. (*D*) LC-MS spectra corresponding to oryzalin and four metabolites. The chemical structures of four metabolites are elucidated by comparing the molecular weight of each metabolite derived from the MS analyses in this study with those derived from oryzalin degradation in soil (26).

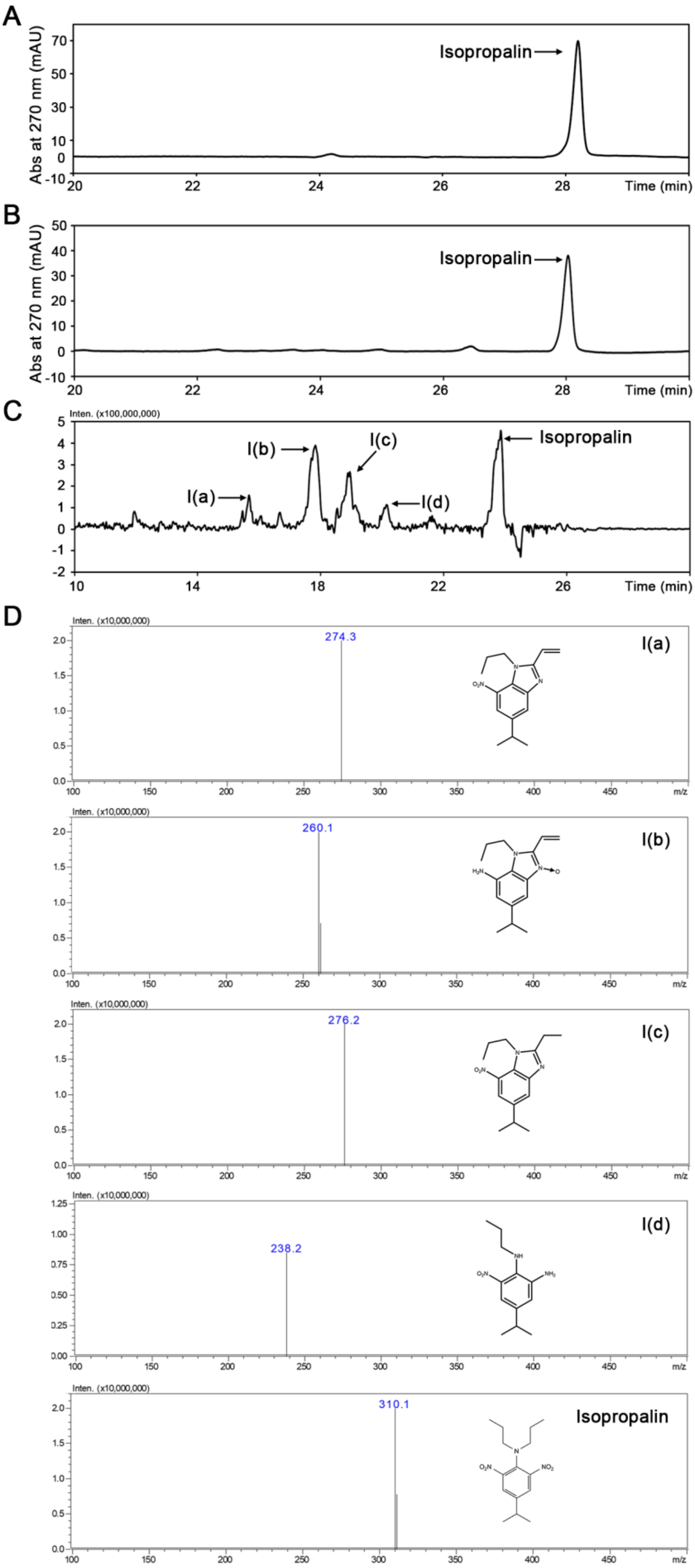

A
Abs at 270 nm (mAU)
Isopropalin
Time (min)
B
Abs at 270 nm (mAU)
Isopropalin
Time (min)
C
Inten. (x100,000,000)
I(a)
I(b)
I(c)
I(d)
Isopropalin
Time (min)
D
Inten. (x10,000,000)
274.3
I(a)
260.1
I(b)
276.2
I(c)
238.2
I(d)
310.1
Isopropalin
m/z

FIGURE S7. LC-MS analyses of metabolites from isopropalin in the Y88A mutant. (*A*) The HPLC chromatogram of isopropalin is shown as a standard. (*B*) The HPLC chromatogram of the Y88A-dependent reaction for 30 min displays a peak for isopropalin at 28.0 min. Other metabolites were not detected at an absorbance of 270 nm. (*C*) TIC identified four metabolites, I(a), I(b), I(c), and I(d). (*D*) LC-MS spectra corresponding to isopropalin and four metabolites. We propose that isopropalin follows a degradation pathway similar to that of oryzalin. The chemical structures of four isopropalin metabolites are elucidated by comparing the molecular weight of each metabolite derived from the MS analyses in this study with those derived from the degradation of isopropalin.

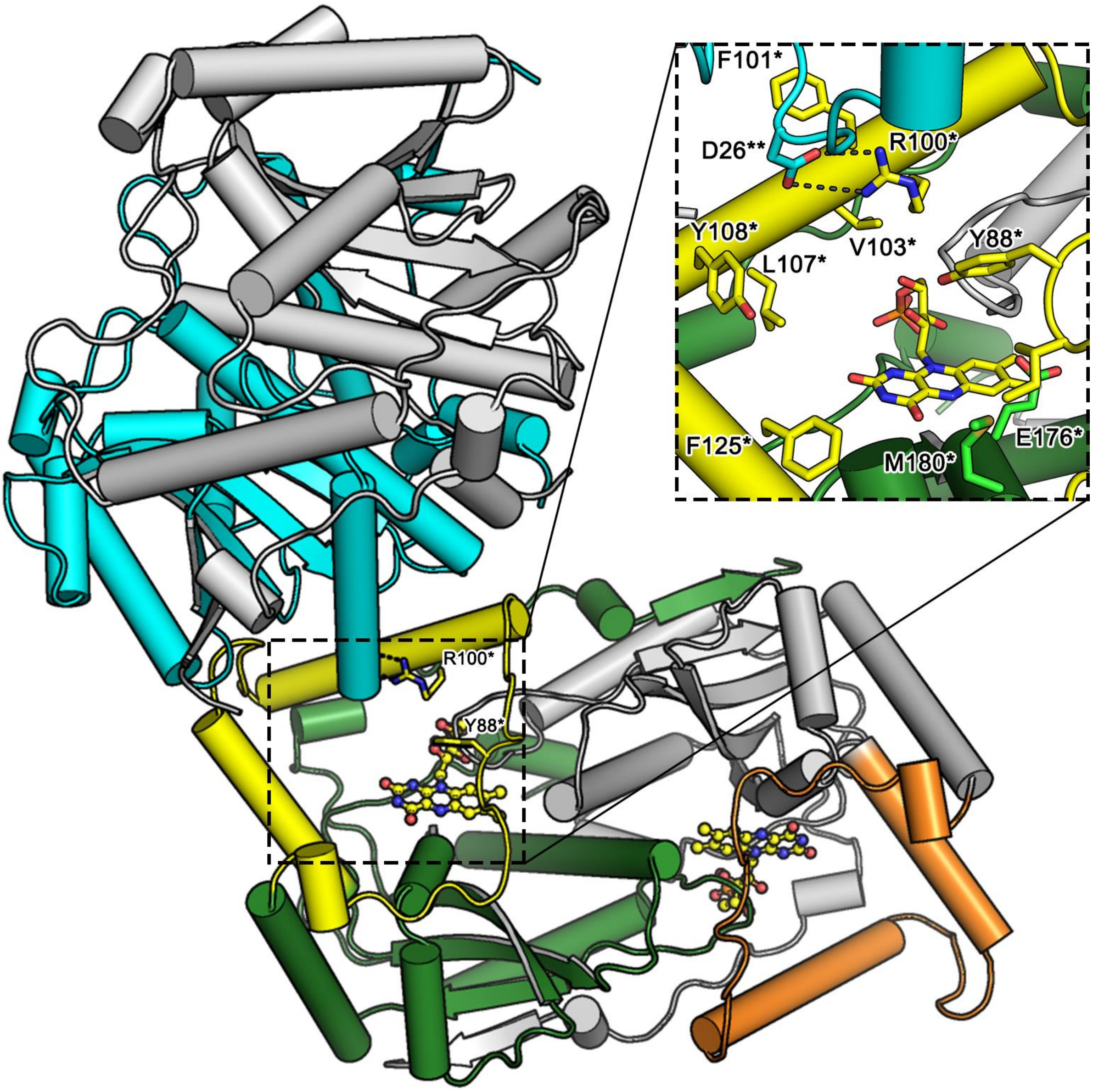

FIGURE S8. Crystallographic packing interactions by Arg100 and Asp26. Crystallographic packing interactions between Arg100 in one of two monomers and Asp26 from a crystallographic symmetry-related monomer. Two dimers under crystalline packing conditions are shown: one dimer with color codes identical to those in Figure 2*A*, and the other dimer with each monomer indicated in gray and cyan, respectively. Note that monomers in green and cyan are equivalent according to crystallographic symmetry. An enlarged view of the crystallographic packing demonstrates that Arg100 has a swung-out conformation for side chain interactions with Asp26**, where the double asterisk indicates a residue from a crystallographic symmetry-related monomer.

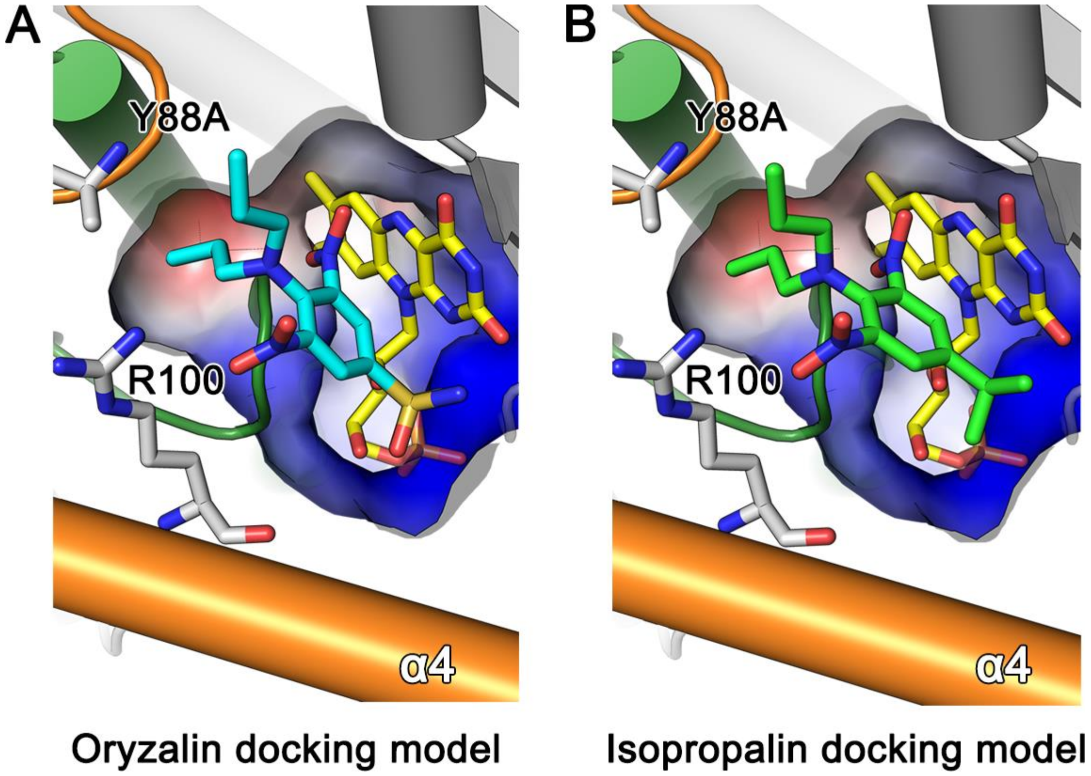

FIGURE S9. Possible binding modes of oryzalin and isopropalin in the active site of the Y88A mutant. The binding modes of (*A*) oryzalin in cyan and (*B*) isopropalin in green were calculated using AutoDock Vina (27), and are shown in an orientation identical to that in Fig. 5C. The predicted binding affinity of oryzalin and isopropalin is –6.9 and –6.4 kcal/mol, respectively; these are the most energetically stable binding modes according to AutoDock. Note that the larger *N*,*N*-dipropyl groups at the amino group in the C1 position, in both oryzalin and isopropalin, are proximal to the Y88A side chain, allowing bulky substrates such as oryzalin and isopropalin to be efficiently catalyzed in the corresponding mutant.

Table S1. Primer sequences used for site-directed mutagenesis

Mutants		Sequence
**NfnB I87A**	Forward Primer	5'-CAGCCCGAATATGAC**GCG**TATCCCCGGGGGCTC-3'
	Reverse Primer	5'-GAGCCCCCGGGGATA**CGC**GTCATATTCGGGCTG-3'
**NfnB Y88A**	Forward Primer	5'-CCCGAATATGACATC**GCG**CCCCGGGGGCTCACC-3'
	Reverse Primer	5'-GGTGAGCCCCCGGGG**CGC**GATGTCATATTCGGG-3'
**NfnB Y88F**	Forward Primer	5'-CCCGAATATGACATC**TTT**CCCCGGGGGCTCACC-3'
	Reverse Primer	5'-GGTGAGCCCCCGGGG**AAA**GATGTCATATTCGGG-3'
**NfnB R100K**	Forward Primer	5'-CCGTGGGACAGCCGC**AAA**TTCGGCGTCGGCGAG-3'
	Reverse Primer	5'-CTCGCCGACGCCGAA**TTT**GCGGCTGTCCCACGG-3'
**NfnB R100A**	Forward Primer	5'-CCGTGGGACAGCCGC**GCG**TTCGGCGTCGGCGAG-3'
	Reverse Primer	5'-CTCGCCGACGCCGAA**CGC**GCGGCTGTCCCACGG-3'
**NfnB R100M**	Forward Primer	5'-CCGTGGGACAGCCGC**ATG**TTCGGCGTCGGCGAG-3'
	Reverse Primer	5'-CTCGCCGACGCCGAA**CAT**GCGGCTGTCCCACGG-3'
**NfnB R100D**	Forward Primer	5'-CCGTGGGACAGCCGC**GAT**TTCGGCGTCGGCGAG-3'
	Reverse Primer	5'-CTCGCCGACGCCGAA**ATC**GCGGCTGTCCCACGG-3'
**NfnB R100E**	Forward Primer	5'-CCGTGGGACAGCCGC**GAA**TTCGGCGTCGGCGAG-3'
	Reverse Primer	5'-CTCGCCGACGCCGAA**TTC**GCGGCTGTCCCACGG-3'
**NfnB F101A**	Forward Primer	5'-TGGGACAGCCGCCGC**GCG**GGCGTCGGCGAGGCG-3'
	Reverse Primer	5'-CGCCTCGCCGACGCC**CGC**GCGGCGGCTGTCCCA-3'
**NfnB V103A**	Forward Primer	5'-AGCCGCCGCTTCGGC**GCG**GGCGAGGCGCTTTAC-3'
	Reverse Primer	5'-GTAAAGCGCCTCGCC**CGC**GCCGAAGCGGCGGCT-3'
**NfnB L107A**	Forward Primer	5'-GGCGTCGGCGAGGCG**GCG**TACGCCTCGCTCGGC-3'
	Reverse Primer	5'-GCCGAGCGAGGCGTA**CGC**CGCCTCGCCGACGCC-3'
**NfnB Y108F**	Forward Primer	5'-GTCGGCGAGGCGCTT**TTT**GCCTCGCTCGGCATC-3'
	Reverse Primer	5'-GATGCCGAGCGAGGC**AAA**AAGCGCCTCGCCGAC-3'
**NfnB Y108A**	Forward Primer	5'-GTCGGCGAGGCGCTT**GCG**GCCTCGCTCGGCATC-3'
	Reverse Primer	5'-GATGCCGAGCGAGGC**CGC**AAGCGCCTCGCCGAC-3'
**NfnB F125A**	Forward Primer	5'-GGGCGGATCGCCCAG**GCG**CAGCAGAATTACCGC-3'

	Reverse Primer	5'-GCGGTAATTCTGCTG**CGC**CTGGGCGATCCGCCC-3'

The mutated sequences are underlined.
